# Effects of inspiratory muscle training on 1RM performance and body composition in professional natural bodybuilders

**DOI:** 10.3389/fphys.2025.1574439

**Published:** 2025-04-07

**Authors:** Baha Engin Çelikel, Coşkun Yılmaz, Alper Demir, Süreyya Yonca Sezer, Levent Ceylan, Tülay Ceylan, Çetin Tan

**Affiliations:** ^1^ Firat University, Faculty of Sport Sciences, Elazığ, Türkiye; ^2^ Gümüşhane University, Kelkit Aydın Dogan VS, Gümüşhane, Türkiye; ^3^ Munzur University, Faculty of Sport Sciences, Tunceli, Türkiye; ^4^ Hitit University, Faculty of Sport Sciences, Çorum, Türkiye; ^5^ Ondokuz Mayıs University, Graduate Education Institute, Samsun, Türkiye

**Keywords:** inspiratory muscle training, bodybuilding, resistance training, muscle hypertrophy, post-activation performance enhancement, physical performance

## Abstract

**Background:**

The effect of inspiratory muscle training on upper limbs One Rep Maximum (1RM) in professional natural bodybuilders is still unclear. Our aim of this study is to investigate the effects of a 6 weeks inspiratory muscle training on 1RM results.

**Methods:**

This study included 14 athletes, that had participated in international bodybuilding competitions. Each athlete had been training for minimum of 5 years and at least 5 h per week. The participants were randomly divided into two groups: inspiratory muscle training (IMT) and control (CON) groups. The IMT group and CON group consisted of seven subjects. While the control group continued with the normal training regime, the IMT group additionally performed inspiratory muscle training with the resistance setting of the PowerBreathe® Classic device set to 40% of the participant’s MIP. Prior to and during the 6-week training period, the bench press 1 RM, anthropometry, pulmonary function tests, and maximal inspiratory (MIP) and expiratory (MEP) pressure measurements were obtained. A nutrition protocol developed by a qualified dietician was implemented for each athlete.

**Results:**

The mean maximal strength of the respiratory muscle training group changed by 14.39%, whereas that of the control group changed by 9.43% (p = 0.012). The changes in the mean FVC (p = 0.000), FEV1 (p = 0.001), PEF (p = 0.064), MIP (p = 0.001), and maximal expiratory pressure (p = 0.017) were greater in the IMT group. The mean circumferences of the shoulder (p = 0.004), chest (p = 0.008), arm (p = 0.004), and neck (p = 0.003) improved more in the IMT group than in the CON group. A reduction in abdominal measurement was observed in the IMT group (p = 0.039), whereas no notable discrepancy was identified in body fat percentage (p = 0.295).

**Conclusion:**

In conclusion we identified that the addition of progressive loading inspiratory muscle training for competition preparation programs in professional natural bodybuilders will further improve their respiratory function, respiratory muscle strength, maximal strength, and muscle development. This research provides valuable insights into how IMT influences bodybuilding training outcomes, potentially guiding future interventions and program designs to better support strength development and body composition in bodybuilding training contexts.

## 1 Introduction

Bodybuilding athletes often perform strength training programs involving muscle work against high resistance at least 3 days a week, aiming to increase muscle mass (Türkmen and Pinarli, 2022). Since inhaling and exhaling becomes difficult during weight lifting in bodybuilding training, it activates the respiratory muscles by providing a stimulus similar to respiratory muscle training (Brown et al., 2013; Escalante et al., 2021). In studies conducted on this type of training, the functional residual capacity (FRC) does not increase (DePalo et al., 2004). However, respiratory muscle forces increase lung parameters by increasing the maximum inspiratory pressure effort (MIP), maximum expiratory pressure effort (MEP), vital capacity (VC), and total lung capacity (TLC) (Brown et al., 2013; Enright et al., 2006).

The respiratory muscles are highly utilized during weight lifting by performing the Valsalva maneuver (VM), which has the same value as expiratory pressure or mouth pressure (Niewiadomski et al., 2012). The Valsalva manoeuvre (VM) is a technique that involves forcing the individual to expel air forcefully and abruptly while the glottis is closed. This sudden and forced expulsion triggers substantial alterations in the cardiovascular system (CVS), particularly in terms of heart rate (HR) and blood pressure (BP), resulting in a series of mechanical and autonomic reflex effects. The individual’s cardiac function undergoes adjustments in response to fluctuations in effective central blood volume and blood pressure, thereby affecting blood flow throughout the body. A lack of respiratory muscle strength negatively affects performance and can lead to possible injury (Blazek et al., 2020). Respiratory muscle strength indicates the ability of inspiratory or expiratory muscles to generate force during a short quasistatic contraction. Although inspiratory muscle strength is important for aerobic performance (Ozmen et al., 2017), bodybuilding training with a low number of repetitions utilizes respiratory and abdominal muscles. Furthermore, the respiratory and abdominal muscles play crucial roles in generating intra-abdominal pressure, which is important in overcoming resistance during strength exercises that involve the development of the upper limbs and anterior torso, such as the bench press (Blazek et al., 2020). When performing each of the techniques required for this movement, the generation of intra-abdominal pressure is noteworthy. It occurs through contraction of the respiratory muscles and diaphragm (DePalo et al., 2004; Al-Bilbeisi and Dennis, 2000).

During bodybuilding training, the diaphragm is recruited alongside other respiratory muscles, contributing to trunk stabilization and providing a stimulus for increased respiratory muscle strength (Hackett et al., 2013). This is particularly crucial during exercises with elevated axial loading, where enhanced support is required as lifting loads increase. Trunk stabilization is achieved through intra-abdominal pressure, which has been observed to rise proportionally with inspiratory respiratory capacity during both the Valsalva maneuver (VM) and breath holding. These core stability mechanisms serve as a stimulus for the observed improvements in respiratory muscle strength. Accordingly, a positive correlation has been reported between respiratory muscle strength measured via maximal inspiratory pressure (MIP) and maximal expiratory pressure (MEP) and maximal lifting performance in bodybuilding-trained athletes (Hackett et al., 2013).

Studies examining the effects of bodybuilding training on respiratory function have reported no significant changes in functional residual capacity (FRC) (DePalo et al., 2004). However, improvements in MIP and MEP have been associated with enhancements in vital capacity (VC) and total lung capacity (TLC) (Brown et al., 2013; Enright et al., 2006). Additionally, expiratory and inspiratory muscle strength have been shown to influence weightlifting performance (Pipat and Weerapong, 2008; Hackett and Sabag, 2021).

Inspiratory muscle training (IMT) is a widely used method for strengthening the inspiratory muscles and improving exercise tolerance (Fernández-Lázaro et al., 2021; Kowalski et al., 2023; Illidi et al., 2023). The physiological benefits of IMT are primarily attributed to the delay or attenuation of the respiratory metaboreflex (Illidi et al., 2023; Kowalski et al., 2023; Fernández-Lázaro et al., 2023). Increased fatigue and metabolite accumulation in the respiratory muscles lead to a redistribution of blood flow from skeletal muscles to respiratory muscles, resulting in reduced perfusion to exercising limbs (Sheel et al., 2018). Consequently, exercise-induced vasoconstriction in active muscles may exacerbate local fatigue and ultimately limit performance (McConnell and Lomax, 2006; Romer et al., 2006). By enhancing the mechanical efficiency and fatigue resistance of the respiratory muscles, IMT is expected to mitigate the accumulation of exercise-induced muscle metabolites and attenuate their systemic effects (Sheel et al., 2018; Illidi et al., 2023; Kowalski et al., 2023).

IMT has been implemented to minimize or delay respiratory fatigue, mitigate the metabolic reflex mechanism of respiratory muscles, and reduce blood lactate accumulation (Edwards and Cooke, 2004; Archiza et al., 2018). As such, IMT has the potential to serve as an ergogenic aid capable of enhancing athletic performance (McConnell, 2005; Fernández-Lázaro et al., 2021; Fernández-Lázaro et al., 2023; Kowalski et al., 2023). Moreover, IMT has been shown to induce physiological adaptations, including diaphragm hypertrophy, increased blood flow to locomotor muscles, reduced fatigue, decreased dyspnea, enhanced respiratory efficiency and endurance, shifts in muscle fiber composition favoring type I fibers and an increase in type II fibers in the intercostal muscles, optimization of neuromotor control in respiratory muscles, and the maintenance of pressure production with minimal motor stimulus (Salazar-Martínez et al., 2017; Kowalski et al., 2023; Fernández-Lázaro et al., 2023).

Although the relationship between the respiratory system and muscle strength is well established, the specific effect of inspiratory muscle training (IMT) on one-repetition maximum (1RM) performance in natural bodybuilders who do not use anabolic steroids remains uncertain. The central hypothesis of this study is that IMT will enhance 1RM performance in bodybuilders by improving non-respiratory muscle function. In light of this hypothesis, the present study aimed to investigate the effects of 6 weeks of inspiratory muscle training on 1RM performance in natural bodybuilders.

## 2 Materials and methods

### 2.1 Participants

The study included 14 male athletes with an average of 5.4 ± 1.2 years of experience in bodybuilding who had engaged in more than 5 h of training per week and had participated in national and international professional natural bodybuilding competitions. The study was designed as a randomized, controlled experimental study. The participants were randomly assigned to two distinct groups: the IMT group and the CON group. The GPower 3.1 program was employed to ascertain the requisite number of participants. The results of the power analysis sampling study indicated that the study could be completed with seven subjects in each group (effect size: 0.80; actual power: 0.89). To determine which group the subjects forming the sample would be included in, the numbers from 1 to 14 were randomly assigned to two groups through a computerised program (https://www.randomizer.org/). Seven natural bodybuilders (age = 23) were selected for the study. The IMT group comprised seven subjects with a mean age of 23.29 ± 2.98 years, a mean height of 181.86 ± 5.24 cm, a mean weight of 84.07 ± 7.35 kg, and a mean body mass index (BMI) of 25.41 ± 1.63 kg/m^2^. The control group consisted of seven subjects with a mean age of 22.57 ± 1.51 years, mean height of 180.57 ± 5.22 cm, mean weight of 80.29 ± 5.41 kg, and mean BMI of 24.89 ± 0.97 kg/m^2^. To avoid any potential confounding effects of the dominant hand and strength on the results, only individuals with a dominant right hand were included in the study **(** Evyapan and Karahan, 2023). In the study, all participants underwent the same training program to exclude the contralateral effect (Manca et al., 2017). Individuals were excluded from the study if they did not meet the following criteria: they were not professional natural bodybuilders, had less than 5 years of experience, had not participated in national or international competitions, or had a chronic disease. Prior to the commencement of the study, all participants were required to provide verbal and written informed consent.

### 2.2 Experimental design

The natural bodybuilders who participated in the study were required to visit the laboratory environment three times. During the initial visit, the experimental procedures were introduced and tested. Each subject was provided with a detailed explanation of the IMT procedure. On the subsequent visit, which occurred 1 week later, pre-workout measurements were taken, and the values were recorded. At the conclusion of the 6-week training program, the final measurements were taken during the third and final visits.

### 2.3 Body composition measurement

Gaia 359 Plus Body-pass bioelectrical impedance analyzer was used to measure the body composition of the athletes who visited the laboratory of Gümüşhane University, Faculty of Sport Sciences. This device employs a measurement method that produces and calculates information about the tissue according to the type of resistance encountered by low electrical currents as they move through body tissues. The Gaia 359 Plus BodyPass was used to determine the subject’s height, body weight, body mass index (BMI), and body fat percentage. The subjects were instructed to stand on the analyzer with the entire sole of the bare foot in contact and to remove any outer garments, including t-shirts and shorts. The subjects were instructed to remove any metallic objects prior to the commencement of the measurement.

Environmental measurements were also taken. Perimeter measurements were taken with a nonelastic, 7 mm wide flexible tape measure while the subject was standing in thin clothing and recorded in centimeters. The data were recorded in centimeters. Circumference measurements were conducted on the chest, neck, arms, abdomen, and shoulders in accordance with the methodologies recommended by the International Society for the Development of Kinanthropometry (Norton and Olds, 1996).

### 2.4 Pulmonary function tests

Peak expiratory flow maximum (PEFmax), Forced expiratory volume in first second (FEV_1_), FEV_1_/FVC (Tiffenau index), and Forced vital capacity (FVC) capacities were analyzed with an MGF Diagnostics CPFS/D USB (Saint Paul, Minnesota, United States) spirometer. Individuals with a FEV1/FVC value <75%, with any chronic or acute disease, using drugs that may affect lung function, or with a history of upper respiratory tract infection were excluded from the study. Pulmonary function measurements were performed while the patients were standing. During the tests, the subjects wore a nose clip to prevent air from escaping and were instructed to keep their lips tightly around the mouthpiece piece (American Thoracic Society, 2002).

### 2.5 Maximal inspiratory (MIP) and expiratory (MEP) pressure measurements

The MIP and MEP were measured with a portable hand-held oral respiratory pressure meter (MicroRPM, CareFusion Micro Medical, Kent, United Kingdom) according to the guidelines of the American Thoracic Society and European Respiratory Society (ATS/ERS, 2002). After the appropriate filters and holders were secured, the nasal airway was closed with a clip. The mouthpiece assembly included a 1 mm hole to prevent glottic closure and minimize the contribution of the cheek muscles during inspiratory efforts. Inspiratory and expiratory maneuvers were performed while standing, with MIP and MEP measurements starting with residual volume and total lung capacity, respectively, and continued for at least 1 s. The measurements were repeated until there was a 5% difference between the two best findings, and the results were recorded in cm H_2_O (Polkey et al., 1995).

### 2.6 Rate of perceived exertion (RPE)

The RPE is a subjective method of measuring an individual’s perception of the physical demands of a given activity. The most commonly utilised RPE instrument is the Borg scale, a psychophysical, categorical scale with a rating ranging from 6 (no exertion) to 20 (maximum exertion) (ACSM, 2010).

### 2.7 Inspiratory muscle training (IMT)

A POWERbreathe® (POWER® Breathe Classic, IMT Technologies Ltd., Birmingham, United Kingdom) device was used for inspiratory muscle training (Fernández-Lázaro et al., 2021; Fernández-Lázaro et al., 2023). IMT was performed for 6 weeks, 6 days a week, twice a day (morning and evening at the same time of day). In each training session, the participant performed 30 breathing cycles (60 breathing cycles per day) (Kilding et al., 2010). This procedure was chosen because it has been previously studied in healthy individuals (Karsten et al., 2019). The resistance setting of the POWERbreathe® device for the IMT was adjusted to 40% of the participant’s MIP (Kantarson et al., 2010). It was increased by 10 cmH2O (1 unit) weekly (Bostanci et al., 2019). Each subject’s morning and evening IMT training sessions were controlled and performed by an expert coach (Alper Demir). The experimental group performed IMT applications in addition to the training program determined by the coaches during the precompetition preparation period, whereas the control group continued only the training program.

### 2.8 Weekly training program

In the context of competitive bodybuilding, the preseason period commences 20–12 weeks prior to the competition. The primary objective during this phase is to achieve a minimal level of body fat reduction (Kistler et al., 2014). The RT program and protocols were developed in accordance with the recommendations of the National Strength and Conditioning Association (NSCA) for optimal athletic development. The training programs for both groups were organised by an experienced senior coach and commenced 20 weeks prior to the international competition. The training was conducted 6 days per week, with a maximum interval of 45 s for each exercise and a 30-s inter-set break time (Table 1). A 5–10-min warm-up routine, comprising a variety of dynamic movements, was conducted prior to each training session.

**TABLE 1:** Weekly training program.

1, day	Chest	Shoulder	Triceps
2, day	Back	Biceps	Arm
3, day	Leg	ABS	—
4, day	Shoulder	Chest	Triceps
5, day	Back- Trapeze	Biceps	Arm
6, day	Leg	ABS	—

The exercises were varied on a weekly basis to prevent the onset of monotony. The training program commenced with a weight corresponding to 50% of the maximal strength and four sets for each movement. The weight was subsequently increased by 25% for sets three and four. For each participant, the weights used were recorded during the course of the training. Three days per week, a 30-min session of cardiovascular exercise was conducted. On days dedicated to leg training, a 20-min warm-up on the elliptical trainer preceded the main workup (Table 2). Following the completion of workouts that did not include leg days, a 20-min cycling session was conducted as a form of restorative exercise (Hackett, 2022).

**TABLE 2:** Activities performed for body parts.

1, day	2, day	3, day	4, day	5, day	6, day
Inc.Bench. Press Smith Mac	Lat Pulldown	Leg Extension	SH. Press Mac.	Close Grip Pulldown	Leg Extension
Inc. Db. Fly	Wide Long Pull	Wide Leg Press	DB. Lateral Raise	Low Row	Wide Leg Press
DB. Bench Press	T-Bar Rowing	Leg Curl	Rope Face Pull	Rope Cabel Pullover	Leg Curl
Cabel Crossover	Deadlift	Adductor	Arnold DB. Press	Barbell Row	Adductor
Dumbbell Press	Rope Long Pull	Calf Raise	Chest Press Mac.	Hyperextension	Calf Raise
Dumbbell Front Raise	Barbell Shrug	Crunch	Inc. Cabel Crossover	Uprıght Row	Crunch
Wide Barbell Upright Row	Dumbbell Curl	Leg Hip Raise	Push-Up DB Fly	Z Bar Curl	Leg Hip Raise
Cabel Push Down	Z Bar Scott Curl	Up Side Plank	Cabel Rope	DB Hummer Curl	Up Side Plank
Dumbbell Kick Back	Rope Hummer Curl	Plank (Max Sn.)	Z Bar Overhead Extension	DB Concent Curl	Plank (Max Sn.)
-	Reverse Barbell Curl	-	Reverse Push Down	Reverse Barbell Wrist Curl	-

### 2.9 1 RM estimation method (bench press)

In recent years, to avoid the drawbacks of the 1RM test, prediction equations have been used to determine the maximum force. Most of the existing equations are based on the principle that they work best when a load that will produce a range of 2–10 repetitions is used (Mayhew et al., 1995). The starting load for the 1RM test was defined as the weight that the athletes could or thought they could lift on the basis of their previous experience for not fewer than two repetitions. Bench press exercise was used because of the relationship between the respiratory system and upper extremities (Sobierajska-Rek et al., 2022). Bench press (BP) exercise was used for the 1RM test in our study (the arm angle on a flat bench was 90°, and the angle between the arm and the chest was determined to be 45°). The bench press grip width was measured according to International Powerlifting Federation (IPF) standard lifting practices. The 1RM test was performed with a V/0/V/0 (voluntary tempo: VOL) movement tempo (Wilk et al., 2020). Wrist straps were not allowed to be worn for weight lifting in the study. Each participant was supported with verbal encouragement during their 1RM performance. Mayhew’s formula was used to estimate the bench press 1RM from the load (kg) and number of repetitions completed, as it provides lower absolute errors (Pérez-Castilla et al., 2020).

$$\text{Mayhew}\;\text{Formula}:\left(1\text{RM}=\left(\text{load}/52.5+41.9^{.e\;‐0.055}\;.\text{repetitions}\right)/100\right)$$

### 2.10 Nutrition protocol

Male bodybuilders utilise energy availability (EA) >25 kcal/kg lean mass (FFM) to maintain muscle mass during contest preparation and avoid negative health consequences against lower levels of energy availability (Fagerberg, 2018). Meals during the day were taken at 3.5-h intervals. The average daily caloric intake of the subjects was calculated as 3,750–4,500 kcal (Kim et al., 2011). A diet program was prepared for bodybuilders with 64.2% of calories from cho, 27.1% from protein, and 8.7% from fat. The average protein intake was 3.0 g/kg/day in the bodybuilders. The dietary intake of micronutrients was not adequate for the study subjects. There is moderate evidence that higher protein intake (≥3.0 g/kg/day) promotes improvements in body composition (Ribeiro et al., 2019).

### 2.11 Statistical analysis

Statistical analyses were performed *via* SPSS (Version 21.0 for Windows, Chicago, IL, United States) software, with the statistical significance set at 0.05. The Shapiro‒Wilk normality test was performed to determine the homogeneity of the sample. The pre-test and post-test differences of each group were determined *via* the paired comparison test (paired t-test), and the post-test and pre-test difference values were determined *via* one-way analysis of variance. In addition, in the comparison of paired groups, the effect size was calculated according to Hedges’ g (Hedges, 1981). Moreover, it was interpreted as follows: 0–0.19 insignificant, 0.20–0.59 small, 0.6–1.19 moderate, 1.20–1.99 large, and ≥2.00 very large.

## 3 Results

The mean values for the participants in the respiratory muscle training group who participated in the study were as follows: height, 181.86 ± 5.24 cm; body weight, 84.07 ± 7.35 kg before training, 93.93 ± 10.79 kg after training; age, 23.29 ± 2.98 years; and BMI, 25.41 ± 1.63 kg/m^2^ before training, 28.33 ± 2.19 kg/m^2^ after training. In the control group, the mean height was 180.57 ± 5.22 cm, the mean body weight was 80.29 ± 5.41 kg before training and 89.57 ± 5.35 kg after training, the mean age was 22.57 ± 1.51 years, and the mean BMI, was 24.89 ± 0.97 kg/m^2^ before training and 27.77 ± 1.06 kg/m^2^ after training (Table 3).

**TABLE 3:** Descriptives.

	Training (n:7)		Control (n:7)	
	*X*	S.D	*X*	S.D
Height (cm)	181.86	5.24	180.57	5.22
Weight pre (kg)	84.07	7.35	80.29	5.41
Weight post (kg)	93.93	10.79	89.57	5.35
Age (year)	23.29	2.98	22.57	1.51
BMİ pre (kg/m^2^)	25.41	1.63	24.89	0.97
BMİ post (kg/m^2^)	28.33	2.19	27.77	1.06

A comparison of the pre-training and post-training difference values of the maximal strength (1RM) of the bodybuilders participating in the study revealed a 14.39% improvement in the respiratory muscle training exercise group and a 9.43% improvement in the control group (p = 0.012) (Figure 2a). According to the Borg scale, both groups improved following training; however, the respiratory muscle training group demonstrated superior outcomes (*p* = 0.002, *e. s.,* = 1,695) (Table 4) (Figure 2b).

**TABLE 4:** Comparison of pre-post 1RM and BORG mean difference values.

		n.	*X*	S.D	%	e.s	T	p. (welch)
1RM	Training	7	15.67	4.74	14.39	1.695	10.054	0.012*
	Control	7	9.43	2.15	9.43			
BORG	Training	7	−3.58	0.76	%20.7	0.006	3.980	0.002*
	Control	7	−1.72	0.65	%9.95			

** p < 0,001, * p < 0,05, e.s, effect size, One-Repetition Maximum (1RM), perceived exertion scale (BORG).

**FIGURE 1:**
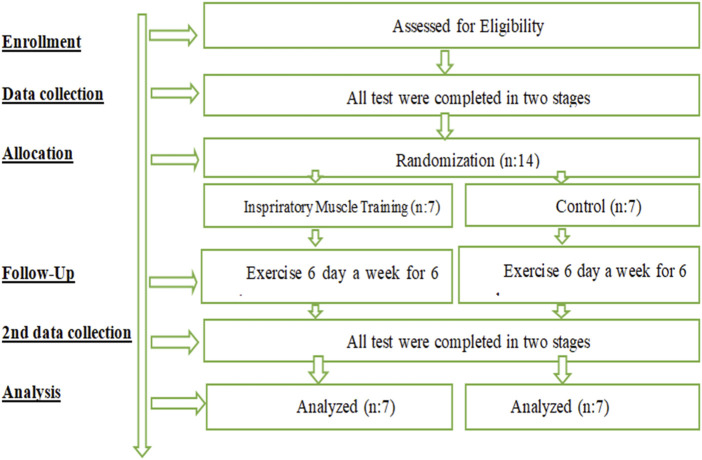
Experimental design.

**FIGURE 2:**
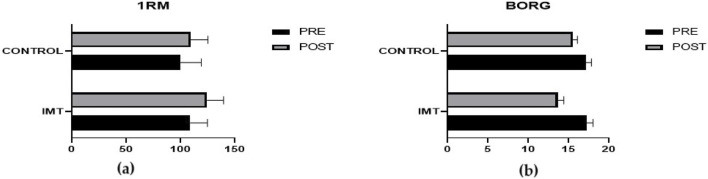
Comparison of pre-post 1RM **(a)** and BORG **(b)** mean difference values.

**FIGURE 3:**
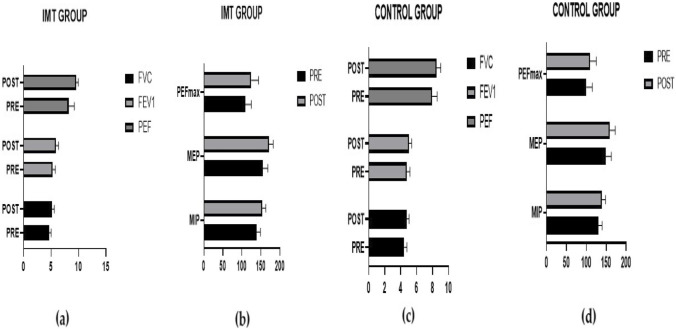
Comparison of pre- and post-training lung function and muscle strength differences between the IMT group **(a, b)** and the control group **(c, d)**.

Greater changes were found in the FVC (p = 0.000), FEV1 (p = 0.001), PEF (p = 0.064), MIP (p = 0.001), and MEP (p = 0.017) before and after 6 weeks in the IMT group (Table 5) (Figure 3).

**TABLE 5:** Comparison of pre- and post pulmonary function and muscle strength difference values.

		n.	*X*	S.D	%	e.s	F	p. (welch)
FVC	Training	7	0.56	0.03	12.07	3.641	44.560	0.000**
	Control	7	0.34	0.08	7.65			
FEV1	Training	7	0.59	0.16	11.09	2.731	25.014	0.001*
	Control	7	0.26	0.06	5.44			
PEF	Training	7	1.37	0.94	16.73	1.180	4.860	0.064
	Control	7	0.56	0.24	7.04			
MIP	Training	7	14.71	2.93	11.33	6.569	23.077	0.001*
	Control	7	9.00	1.15	6.89			
MEP	Training	7	16.71	5.22	10.81	1.609	9.094	0.017*
	Control	7	10.29	2.14	6.91			

** p<0,001, *p < 0,05, e. s., effect size, Peak expiratory flow maximum (PEFmax), Forced expiratory volume in first second (FEV_1_), FEV_1_/FVC (Tiffenau index), FVC, Forced vital capacity; MIP, Maximal inspiratory pressure; MEP, Maximal expiratory pressure.

**FIGURE 4:**
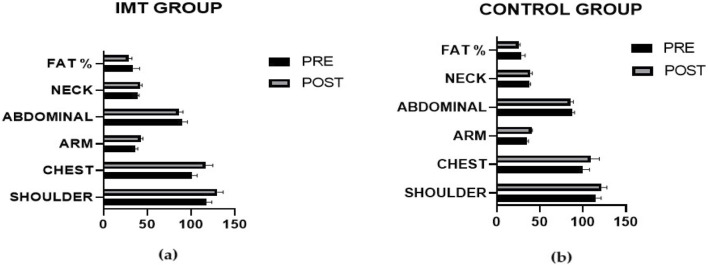
Comparison of pre- and post-intervention differences in body composition between the IMT group **(a)** and the control group **(b)**.

The results of our study indicated that the training group presented greater increases in muscle mass in the shoulder (p = 0.004), chest (p = 0.008), arm (p = 0.004), and neck (p = 0.003) regions than did the control group. A reduction in abdominal measurements was observed in the training group (p = 0.039), whereas no significant difference was detected in the body fat ratio (p = 0.295) (Table 6) (Figure 4).

**TABLE 6:** Comparison of pre-post body composition difference values.

		n.	X	S.D	%	e.s	F	p. (welch)
Shoulder	Training	7	12.29	3.50	10.44	2.009	14.118	0.004*
	Control	7	6.57	1.99	5.70			
Chest	Training	7	15.86	4.53	15.77	1.663	10.651	0.008*
	Control	7	9.29	2.81	9.29			
Arm	Training	7	6.43	0.98	17.93	2.112	15.783	0.004*
	Control	7	4.86	0.38	13.72			
Abdominal	Training	7	−3.86	2.19	−4.31	1.279	5.689	0.039*
	Control	7	−1.57	1.27	−1.81			
Neck	Training	7	3.14	0.90	8.98	2.299	18.615	0.003
	Control	7	1.57	0.35	4.19			
Fat	Training	7	−5.43	3.64	−16.04	0.590	1.213	0.295
	Control	7	−3.57	2.57	−12.43			

** p<0,001, * p < 0,05, e. s., effect size.

## 4 Discussion

The primary objective of the present study was to examine the effects of 6 weeks of IMT on 1RM performance. To the extent of our knowledge, this is the first study to examine the effects of IMT on 1RM performance in bodybuilding. The main findings of this study were that the addition of inspiratory muscle training to the preparation program of professional natural bodybuilders during the competition period improved their respiratory function, respiratory muscle strength, 1RM, and muscle development. Bodybuilding training is known to increase respiratory muscle strength and function (Brown et al., 2013; DePalo et al., 2004), and advanced bodybuilders develop unique respiratory adaptations (Hackett, 2020).

In this study, when the 1RM of the bodybuilders before and after training was compared, the respiratory muscle training group increased by 14.39%, and the control group increased by 9.43%. The respiratory muscle training group affected the 1 RM more in the exercises performed (e.g., = 1.695). In the responses to the Borg Strain Inventory, the respiratory muscle training group had less difficulty after training (p = 0.002).

Jagim et al. (2018) reported that elevation training masks made no significant difference between bench press repetition and total power in recreational bodybuilders. Borujeni and Yalfani (2019) reported that 8 weeks of inspiratory muscle training improved postural control by reducing swing in the overhead and single squat tests. Hackett and Sabag (2021) reported that bench press, squat, and deadlift performance were positively correlated with expiratory muscle strength, but there was no relationship between most lung functions and weightlifting performance.

Since the ventilatory demands of bodybuilding training are generally lower than those of aerobic exercise (Vilaça-Alves et al., 2016), the training stimulus for respiratory muscles differs. The continuous engagement in strength- and endurance-based physical activities induces adaptive changes in spirometric parameters, including vital capacity (VC), forced vital capacity (FVC), and forced expiratory volume in one second (FEV_1_), as well as in respiratory muscle strength (Durmic et al., 2017). Consequently, different sports elicit distinct respiratory adaptations. During bodybuilding training, intra-abdominal pressure increases as a result of heavy loads and accumulated fatigue (Niewiadomski et al., 2012; Hackett, 2020). This increase in intra-abdominal pressure occurs due to diaphragm contraction, which displaces the inspiratory muscle downward and acts upon the relatively incompressible abdominal contents. This process is further supported by the simultaneous activation of the abdominal muscles (Martuscello et al., 2013; Cavaggioni et al., 2015). Therefore, the diaphragm, inspiratory and expiratory respiratory muscles, and abdominal muscles actively contribute to intra-abdominal pressure regulation during bodybuilding training (Hackett, 2020). These maneuvers generate transdiaphragmatic pressures high enough to provide a training stimulus for the respiratory muscles. Repeated exposure to such repeated vigorous weight lifting manoeuvres strengthens the diaphragm and inspiratory and expiratory muscles (DePalo et al., 2004).

Additionally, bodybuilding training is recognized as a potent stimulus for skeletal muscle adaptations, including muscle hypertrophy (Abe et al., 2000). However, the specific impact of inspiratory muscle training (IMT) on bodybuilding performance remains unclear. Lifting heavy loads (>75% of one-repetition maximum) typically activates the Valsalva maneuver, which may explain potential associations between IMT and exercises that require less spinal stability, such as the bench press. Therefore, the adaptations induced by bodybuilding training extend beyond the primary muscles involved in weightlifting exercises and also encompass the respiratory muscles (MacDougall et al., 1992). Increased fatigue and metabolite accumulation in the respiratory muscles during exercise leads to redistribution of blood flow from skeletal muscles to respiratory muscles, resulting in reduced perfusion to the exercising muscles (Sheel et al., 2018). This means that exercise will not be sufficiently efficient (McConnell and Lomax, 2006; Romer et al., 2006). IMT application increases the mechanical efficiency and fatigue resistance of respiratory muscles, reduces the accumulation of exercise-induced muscle metabolites and increases perfusion to the muscles by alleviating systemic effects (Illidi et al., 2023; Kowalski et al., 2023; Fernández-Lázaro et al., 2023). Therefore, IMT is used as an ergogenic aid that can improve athletic performance (McConnell, 2005; Fernández-Lázaro et al., 2021; Fernández-Lázaro et al., 2023; Kowalski et al., 2023). Since measurements related to blood and molecular concepts were not taken in the study, discussions about these concepts will only be speculation.

When we compared the respiratory function and respiratory muscle strength of the bodybuilding athletes included in our study, the respiratory muscle training group presented higher values of FVC (p = 0.000), FEV1 (p = 0.001) and PEF (p = 0.064) values and respiratory muscle strength parameters MIP (p = 0.001) and MEP (p = 0.017). In the literature, IMT has been shown to improve respiratory function and quality of life in patients with chronic obstructive pulmonary disease (Lötters et al., 2002), dyspnea, healthy male smokers (Bostanci et al., 2019), and asthmatics (Duruturk et al., 2018) and to improve respiratory muscle strength, endurance, and exercise performance in athletes (Kilding et al., 2010; HajGhanbari et al., 2013; Hartz et al., 2018; Karsten et al., 2019; Fernández-Lázaro et al., 2021; Fernández-Lázaro et al., 2023; Kowalski et al., 2023).

There is evidence that all types of regular exercise affect spirometric indices and can lead to higher FEV _1_ and FVC values than in sedentary individuals (Myrianthefs et al., 2014; Durmic et al., 2017). These adaptive changes are known to result in athletes having up to 20% greater pulmonary function than sedentary individuals (Cheng et al., 2003). This may be explained by decreased airway resistance, increased alveolar expansion, and increased total lung elasticity caused by regular physical activity. As a result, the strength of the respiratory muscles increases with increasing endurance, as in the control group that only performed bodybuilding training in this study (Durmic et al., 2017).

Bodybuilding training is known to result in numerous positive physiological and functional adaptations (Deschenes and Kraemer, 2002). Muscle strength development and increased muscle mass are two commonly cited changes following bodybuilding training (Folland and Williams, 2007). These adaptations occur in muscles that actively participate in response to the training stimulus, in accordance with the principle of specificity training (Kraemer and Ratamess, 2004). In addition to muscles that are used to produce force directly against an external object, there is also increased activation of muscles that help maintain posture, known as stabilisers (Martuscello et al., 2013). Muscles that help with spinal stability include the diaphragm, chest muscles, respiratory muscles (inspiratory and expiratory), and abdominal muscles (Martuscello et al., 2013). These muscles have a dual role, as they are also involved in the mechanics of breathing (Welch et al., 2019). Therefore, it is not surprising that weightlifters have greater diaphragm mass and respiratory muscle strength than untrained healthy adults do (McCool et al., 1997). The effectiveness of bodybuilding training on respiratory muscle strength has been confirmed in apparently healthy (DePalo et al., 2004) and clinical populations (Menezes et al., 2016). However, improvements in respiratory muscle function after bodybuilding training may be influenced by the type of training program (Al-Bilbeisi and Dennis, 2000). The activation of the respiratory muscles and diaphragm and the weight of the load lifted are important factors. Targeting respiratory muscles in training programs may help improve weightlifting performance (Hackett and Sabag, 2021). In this study, it was found that focusing on respiratory muscles increased 1RM and body composition parameters.

The mean circumferences of the shoulder (p = 0.004), chest (p = 0.008), arm (p = 0.004), and neck (p = 0.003) increased more in the training group than in the control group. There was a decrease in abdominal measurement (p = 0.039) in the exercise group, whereas no significant difference was found in body fat percentage (p = 0.295).

In the study of Bostan and Gümüş (2022), the changes between the pre-test and post-test values of the general body, leg, arm, and trunk measurements of people who participated in fitness training and EMS training did not significantly differ according to the type of training performed. Yue et al. (2018) compared the effects of two different volume-equalised weight training strategies on body composition and performance. Although both training strategies increased performance and decreased body muscle mass, only the high-volume, low-frequency protocol increased upper body hypertrophy and improved body composition. Skeletal muscle can adapt to exercise stimuli because of its mechanical and metabolic properties. These changes have been shown to be specific to the type of exercise stimulus; intense resistance exercise usually increases muscle size and strength, whereas exercise at much lower loads results in an increase in muscle oxidative capacity, although it does not affect muscle size (Takarada et al., 2000; Fry, 2004). IMT application in addition to the training applied in the study increases the perfusion to the muscles because it increases the mechanical efficiency and fatigue resistance of the respiratory muscles (Illidi et al., 2023; Kowalski et al., 2023; Fernández-Lázaro et al., 2023). This means that more oxygen will be delivered to the muscles. Oxygen uptake is known to be the variable that best influences weight and exercise intensity (Vilaça-Alves et al., 2016). Therefore, it is likely that body composition values will increase as the respiratory system develops, which is also the result of the study.

As a limitation of the study, although nutrition programs were provided, the athletes were not followed one-on-one. It was assumed that the participants followed the nutritional programs. In addition, athletes who did not participate in national or international competitions were not included in the study. The number of participants was limited due to the population of elite, high-performance male natural bodybuilders. A larger sample size may provide more accurate data. Detailed applications related to the evaluation of the diaphragm such as ultrasound examination were not performed. Only 1RM and body composition parameters were evaluated as physical assessment.

## 5 Conclusion

The central hypothesis of this study, which proposed that inspiratory muscle training (IMT) would enhance 1RM performance in professional natural bodybuilders by improving non-respiratory muscle function, has been confirmed. Furthermore, respiratory function, respiratory muscle strength, maximal strength, and body composition showed greater improvements in athletes who incorporated IMT into their competition preparation programs compared to those following regular training protocols. Based on these findings, the inclusion of IMT in pre-competition training programs for professional natural bodybuilders is recommended. In addition to IMT, further research utilizing different methodologies and measurement techniques to assess the degree of respiratory muscle hypertrophy will help clarify its effects.

## Data Availability

The raw data supporting the conclusions of this article will be made available by the authors, without undue reservation.
